# Supplementary material for: Reconstructing cancer karyotypes from short read data: the half empty and half full glass
Source: BMC Bioinformatics. 2017 Nov 15;18:488. doi: 10.1186/s12859-017-1929-9 (PMC5688766; doi:10.1186/s12859-017-1929-9)
Supplement: Supplementary file 1 — An example of graphs that represent equivalent yet not identical solutions. (DOCX 60 kb) [file 12859_2017_1929_MOESM1_ESM.docx]

Additional file 1: Figure S1


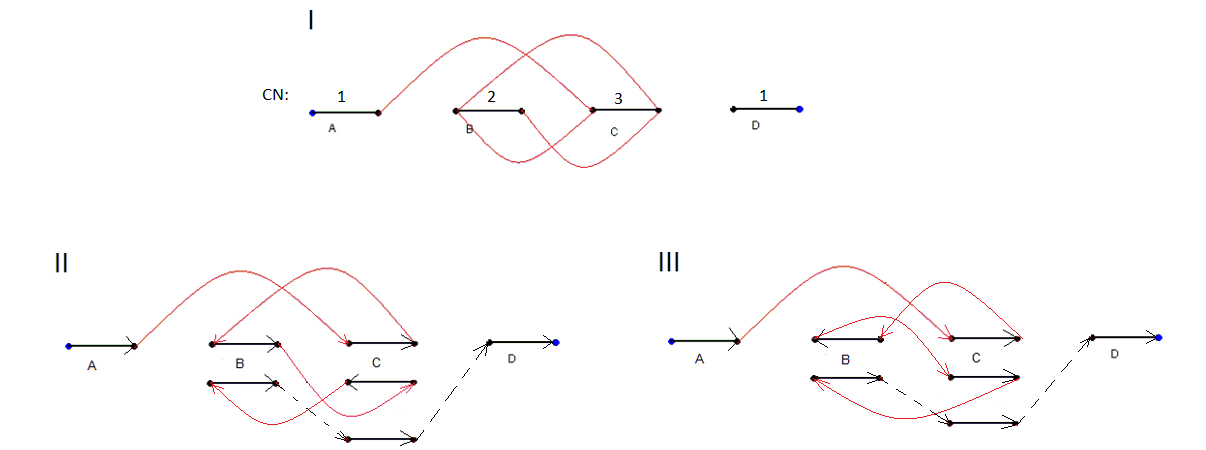


Figure S1: An example of graphs that represent equivalent yet not identical solutions. Graph (I) can have a path of (II) $A\to C\to\boldsymbol{B\to-C}\to B\to C\to D$ or (III)$A\to C\to\boldsymbol{-B\to C}\to B\to C\to D$. Both paths start and end in telomeric nodes (marked blue) and use the exact same set of bridges. In (I) the numbers above the solid edges are CNs. In both alternative paths, the last part of the path is connected by reference edges.
