# Supplementary material for: Reconstructing cancer karyotypes from short read data: the half empty and half full glass
Source: BMC Bioinformatics. 2017 Nov 15;18:488. doi: 10.1186/s12859-017-1929-9 (PMC5688766; doi:10.1186/s12859-017-1929-9)
Supplement: Supplementary file 2 — Edges affected by removing a bridge. (DOCX 66 kb) [file 12859_2017_1929_MOESM2_ESM.docx]

Additional file 2: figure S2


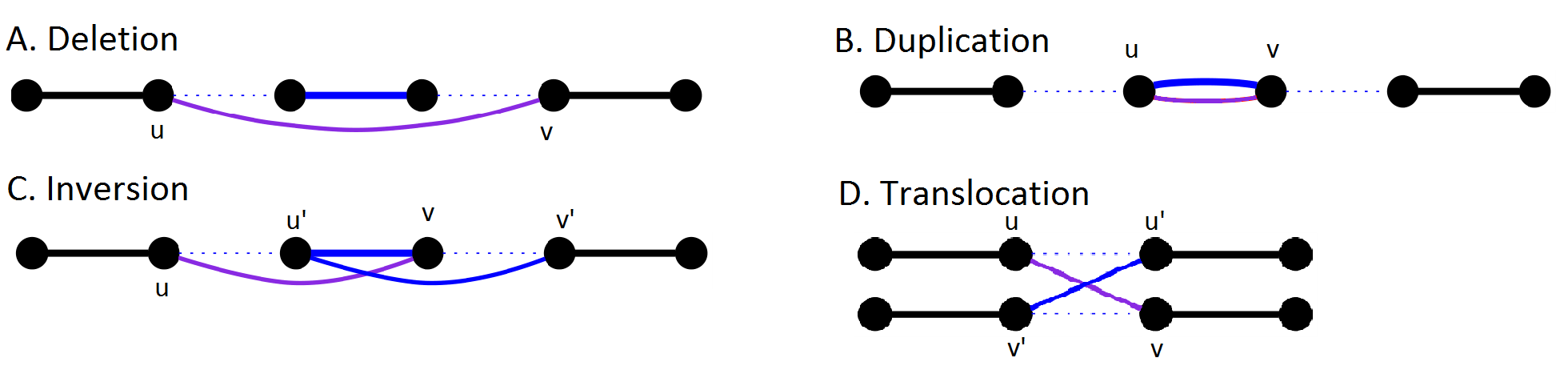


Figure S2: Edges affected by removing a bridge. In each of the four cases, the purple edge $(u,v)$ is the missing bridge and the blue edges are affected by the removal. (A) In the case of deletion, the removal of the bridge will affect all edges in the path$u\to v$. (B) For duplication, the weight assigned to the duplicated segment $\left[ u,v \right]$ will be affected. (C) Inversion creates two bridges. When one is omitted this will affect the score of the other bridge $(u^{'},v^{'})$ and the inverted segment$[u^{'},v]$. (D) A translocation creates two bridges. When the bridge $\left( u,v \right)$ is omitted this will affect the other bridge $\left( v^{'},u^{'} \right)$ and the two corresponding reference edges.
