# Supplementary material for: Reconstructing cancer karyotypes from short read data: the half empty and half full glass
Source: BMC Bioinformatics. 2017 Nov 15;18:488. doi: 10.1186/s12859-017-1929-9 (PMC5688766; doi:10.1186/s12859-017-1929-9)
Supplement: Supplementary file 3 — Boxplot of the distribution for different success measures over 10,000 simulations. (DOCX 30 kb) [file 12859_2017_1929_MOESM3_ESM.docx]

Additional file 3


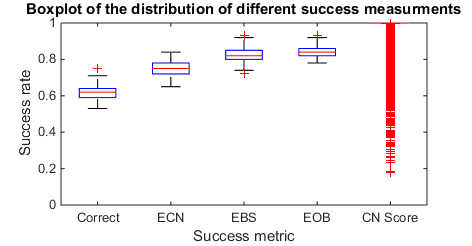


Figure S12: Boxplot of the distribution for different success measures over 10,000 simulations.
