# Supplementary material for: Reconstructing cancer karyotypes from short read data: the half empty and half full glass
Source: BMC Bioinformatics. 2017 Nov 15;18:488. doi: 10.1186/s12859-017-1929-9 (PMC5688766; doi:10.1186/s12859-017-1929-9)
Supplement: Supplementary file 4 — Performance of the algorithm for different values of the parameter alpha. (DOCX 19 kb) [file 12859_2017_1929_MOESM4_ESM.docx]

Additional file 4: table S1

| **Alpha** | **Correct %** | **ECN %** | **EBS %** | **EOB %** |
| --- | --- | --- | --- | --- |
| 0.00 | 13 | 82 | 85 | 17 |
| 0.01 | 67 | 82 | 85 | 82 |
| 0.02 | 67 | 83 | 86 | 90 |
| 0.03 | 67 | 83 | 86 | 90 |
| 0.04 | 67 | 82 | 85 | 89 |
| 0.05 | 67 | 81 | 84 | 90 |
| 0.10 | 67 | 80 | 83 | 89 |
| 0.25 | 67 | 77 | 81 | 86 |
| 0.50 | 67 | 73 | 77 | 82 |
| 1.00 | 67 | 69 | 73 | 78 |
| 2.00 | 67 | 68 | 72 | 77 |

Table S1: Performance of the algorithm for different values of the parameter alpha.
