# Supplementary material for: Reconstructing cancer karyotypes from short read data: the half empty and half full glass
Source: BMC Bioinformatics. 2017 Nov 15;18:488. doi: 10.1186/s12859-017-1929-9 (PMC5688766; doi:10.1186/s12859-017-1929-9)
Supplement: Supplementary file 5 — Results of different simulated scenarios. (DOCX 33 kb) [file 12859_2017_1929_MOESM5_ESM.docx]

Additional file 5: Figures S3 – S8. Results of different simulated scenarios.

Figure S3: The effect of the number of chromosomes on the success rate of the algorithm.

Figure S4: Performance where the original reference genome is haploid (single copy) and diploid.

Figure S5: Performance as a function of the probability to miss a bridge.

Figure S6: Performance of the algorithm on samples contaminated with a second, different karyotype.

Figure S7: Results for different operation frequencies. The uneven frequencies were based on the observed frequencies in ^46^. The frequencies used are described in Additional file 9: Table S3.

Figure S8: Performance for tumors with different levels of contamination from healthy karyotypes.
