# Supplementary material for: Reconstructing cancer karyotypes from short read data: the half empty and half full glass
Source: BMC Bioinformatics. 2017 Nov 15;18:488. doi: 10.1186/s12859-017-1929-9 (PMC5688766; doi:10.1186/s12859-017-1929-9)
Supplement: Supplementary file 6 — Operations frequencies used in the default scenario and in the alternative scenario. (DOCX 18 kb) [file 12859_2017_1929_MOESM6_ESM.docx]

Additional file 6: table S3

| **Type** | **Uniform** | **Actual Malhotra data** | **Simulations** |
| --- | --- | --- | --- |
| Deletion | 25% | 43% | 40% |
| Duplication | 25% | 38% | 40% |
| Inversion | 25% | 12% | 10% |
| Translocation | 25% | 7% | 10% |

Table S3: operations frequencies used in the default scenario and in the alternative scenario.
