# Supplementary material for: Reconstructing cancer karyotypes from short read data: the half empty and half full glass
Source: BMC Bioinformatics. 2017 Nov 15;18:488. doi: 10.1186/s12859-017-1929-9 (PMC5688766; doi:10.1186/s12859-017-1929-9)
Supplement: Supplementary file 7 — The effect of tumor heterogeneity. The file details the effects of tumor heterogeneity on our simulations model. (DOCX 17 kb) [file 12859_2017_1929_MOESM7_ESM.docx]

Additional file 7: the effect of tumor heterogeneity

We tested the algorithm on simulated data of tumors that are heterogeneous. We first simulated a sample that contains, aside from our tumor karyotype, the normal karyotype in rates of up to 45%. This simulates the situation where the sample is a mixture of normal and tumor cells. Results were slightly better for more homogenous samples, but overall the algorithm was able to achieve a success rate of around 60% even for samples that contain only 55% tumor cells (Additional file 3: figure S8).

We also simulated a scenario where the sample is a mixture of two different mutated karyotypes. The lower frequency karyotype was 0%, 5%, 10%, 15% and 20%. The dominating target karyotype underwent 5 rearrangements (as in the base scenario), while the lesser karyotype underwent an average of 3, about half of them unique and the rest are shared between the two. While this is not a strict evolutionary model, it mimics the situation where different karyotypes in the same tumor share some similarities. As expected, this proved to be a more difficult scenario and the rate of correct predictions dropped quickly. The other score metrics exhibited a much slower decline however (Additional file 3: figure S6). Note that the evaluation was done in terms of reconstructing the dominating karyotype only.
