# Supplementary material for: Reconstructing cancer karyotypes from short read data: the half empty and half full glass
Source: BMC Bioinformatics. 2017 Nov 15;18:488. doi: 10.1186/s12859-017-1929-9 (PMC5688766; doi:10.1186/s12859-017-1929-9)
Supplement: Supplementary file 8 — Breakpoint filter. The file describes the details of our breakpoint filter. (DOCX 37 kb) [file 12859_2017_1929_MOESM8_ESM.docx]

Additional file 8: Breakpoint filter

In analyzing real data we discovered that some breakpoints are clustered together in a very small region at close proximity to one another. Since the copy number data has a lower resolution than the paired-end data, this results an unnecessary over-fragmentation of the chromosome into a large number of small fragments that have almost the same measured copy number. To address this, we apply a filter on the list of breakpoints and group together breakpoints that are located very close to one another. The cut-off was set to be 5000 bases – all breakpoint coordinates that are within 5000 bases from each other are treated as the same breakpoint coordinate. This filtering does not throw out the actual bridge data, but ignores rearrangements that are very small and local in nature such as loss of small DNA shards that do not alter the result of the algorithm.

Ignoring the deletion of a small fragment of DNA between intervals $I_{i}, I_{i+1}$ and grouping the relevant breakpoints together creates two nodes $h_{i}, t_{i+1},$ with both variant and reference edges connecting them. As a result the bridge becomes redundant and any resulting path outputted by the algorithm ignores it (Additional file 12: figure S13).


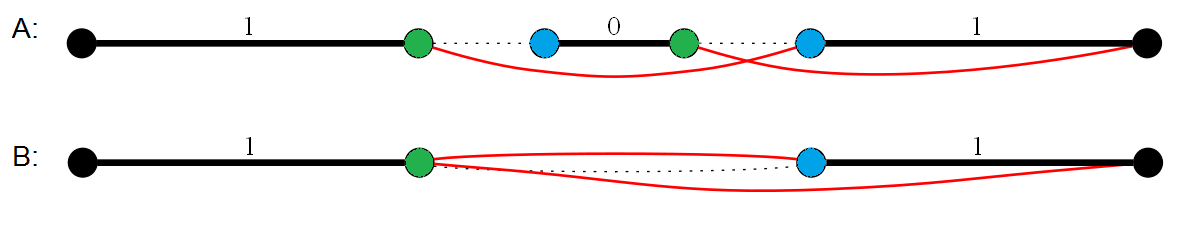


Figure S13: The effect of filtering small intervals. A: The interval adjacency graph including the deletion of a small segment between close breakpoints. B: The graph after applying a breakpoint filter. The blue and green nodes were joined together respectively. After the filtering the original bridge is still included but is rendered redundant and thus ignored by the algorithm.
