# Supplementary material for: Reconstructing cancer karyotypes from short read data: the half empty and half full glass
Source: BMC Bioinformatics. 2017 Nov 15;18:488. doi: 10.1186/s12859-017-1929-9 (PMC5688766; doi:10.1186/s12859-017-1929-9)
Supplement: Supplementary file 9 — Estimation of bridge support in real data. The file details the calculations for estimating bridge support in real data. (DOCX 18 kb) [file 12859_2017_1929_MOESM9_ESM.docx]

Additional file 9: Estimation of bridge support in real data

Recall that the data include bridges and for each bridge an integer value, called *support,* representing the number of paired end reads (PERs) supporting that bridge. The expected average support can be derived from the read depth and the insert size. We assume that in order for a bridge to be supported by a PER, the breakpoint causing it has to fall within the gap of the PER’s insert. In other words, each read of the PER has to be mapped in full to one of the two sides of the breakpoint. Let $ins$ be the total insert length and $end$ be the length of each end, so that the read gap is$gap=ins-2*end$. The depth of coverage is the average number of times a base is sequenced, i.e. covered by one of the ends (as the gap is not sequenced). Equivalently, it is the average time it is covered by an end. Hence, the expected support score for a given breakpoint is$E_{supp}=\frac{d}{\frac{\left( 2*end \right)}{ins}}*\frac{gap+1}{ins}=d*\frac{gap+1}{2*end}$. In the data examined the mean size of each read is$ins=242$, with mean end length of$end=95$. The average coverage is$d=40$, and so the expected support for a given bridge is

$$E_{supp}=40*\frac{242-2*95+1}{2*95}=10.7$$

The observed mean support score across all the data was 10.8. Figure S9 shows the distribution of the support scores across the data. A total of 6170 bridges were reported. Ignoring a few bridges with unusually high support, 6131 bridges (99%) with support score lower than 100 had mean score of 8.63 and standard deviation of 8.44. The support scores across the real data closely resemble an exponential distribution with $\lambda=0.1866$ (Figure 9) the distribution used in our simulation model.
