# Supplementary material for: Reconstructing cancer karyotypes from short read data: the half empty and half full glass
Source: BMC Bioinformatics. 2017 Nov 15;18:488. doi: 10.1186/s12859-017-1929-9 (PMC5688766; doi:10.1186/s12859-017-1929-9)
Supplement: Supplementary file 10 — Histogram of bridge support scores across the data. (DOCX 25 kb) [file 12859_2017_1929_MOESM10_ESM.docx]

Additional file 10: Figure S9


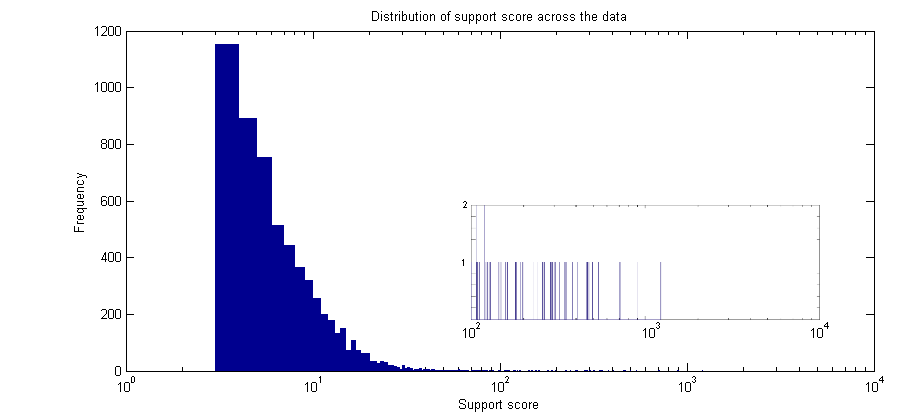


Figure S9: Histogram of bridge support scores across the data. Bridges with support score ≤2 are not included in the data. The inlaid plot shows the distribution of the support scores with values >100.
