# Supplementary material for: Reconstructing cancer karyotypes from short read data: the half empty and half full glass
Source: BMC Bioinformatics. 2017 Nov 15;18:488. doi: 10.1186/s12859-017-1929-9 (PMC5688766; doi:10.1186/s12859-017-1929-9)
Supplement: Supplementary file 11 — Components from the Malhotra data [46] studied by the algorithm. (DOCX 18 kb) [file 12859_2017_1929_MOESM11_ESM.docx]

Additional file 11: table S2

| **Sample** | **Chromosomes comprising the component** | **Number of bridges** | **Number of CNV’s** |
| --- | --- | --- | --- |
| LUAD_6 | 1 | 8 | 40 |
| GBM_10 | 4, X | 7 | 40 |
| LUSC_5 | 6, 12, 15, 16 | 8 | 28 |

Table S2: Components from the Malhotra data ^46^ studied by the algorithm.
