# Supplementary material for: Reconstructing cancer karyotypes from short read data: the half empty and half full glass
Source: BMC Bioinformatics. 2017 Nov 15;18:488. doi: 10.1186/s12859-017-1929-9 (PMC5688766; doi:10.1186/s12859-017-1929-9)
Supplement: Supplementary file 12 — Results on real samples LUAD 6, LUSC 5. (DOCX 314 kb) [file 12859_2017_1929_MOESM12_ESM.docx]

Additional file 12: Results on real samples LUAD 6, LUSC 5.


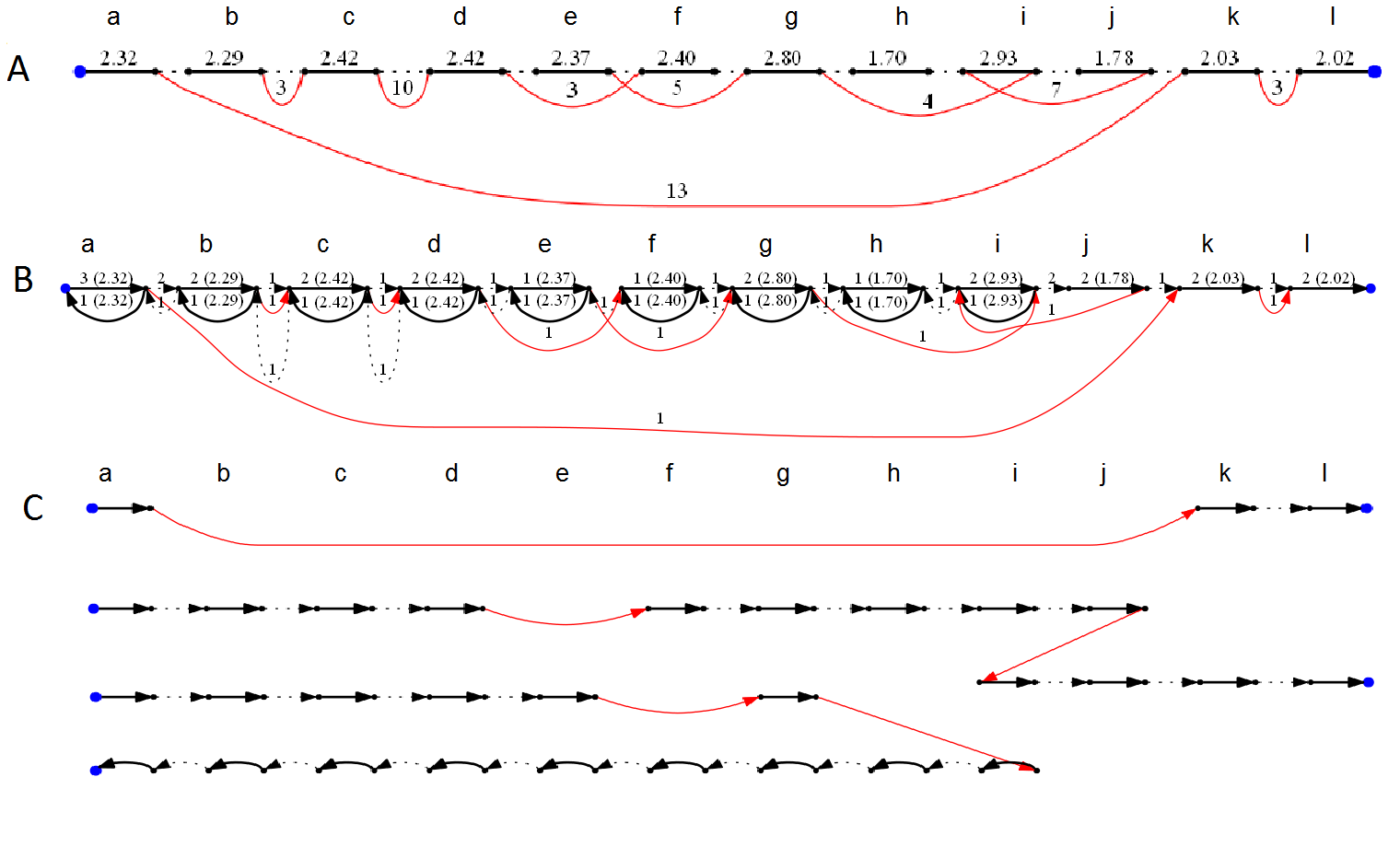


Figure S10: Results of sample LUAD 6. (A) Bridge graph for chromosome 1. (B) Solution suggested by our algorithm. For this sample the average distance of the resulting karyotype from the data, weighted by segment length, is 0.24. (C) The different paths comprising the solution, representing the rearranged karyotype of chromosome 1. This figure was automatically drawn by the graphical software GraphViz ^47^ using the output of the algorithm.


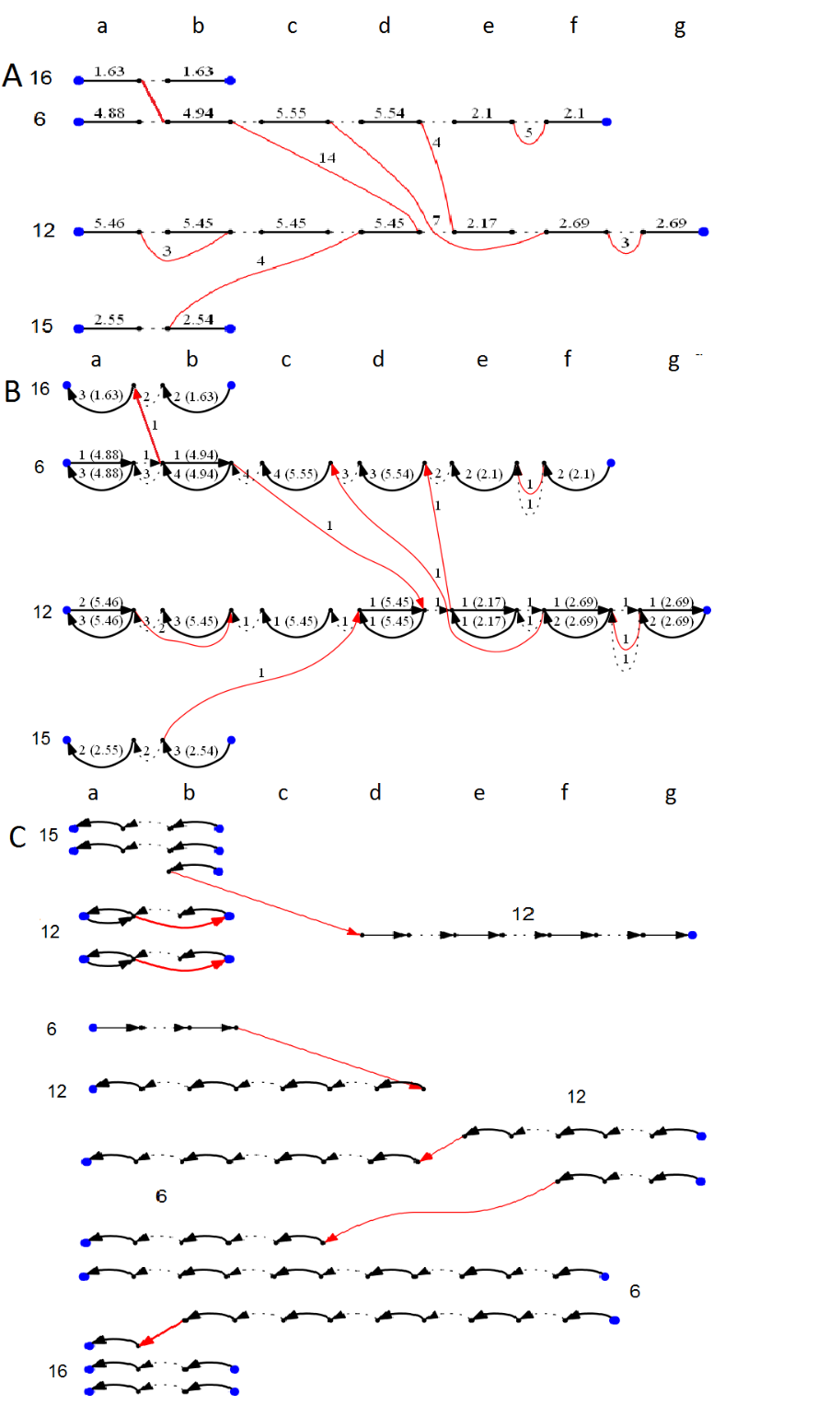


Figure S11: Results of sample LUSC 5. (A) Bridge graph for chromosomes 6, 12, 15, 16. (B) Solution suggested by our algorithm. For this sample the average distance of the resulting karyotype from the data, weighted by segment length, is 0.24.(C) The different paths comprising the solution, representing the rearranged karyotype of chromosomes 6, 12, 15, 16. This figure was automatically drawn by the graphical software GraphViz ^47^ using the output of the algorithm.
